# Lung mechanics during video-assisted abdominal surgery in Trendelenburg position: a cross-sectional propensity-matched comparison between classic laparoscopy and robotic-assisted surgery

**DOI:** 10.1186/s12871-022-01900-5

**Published:** 2022-11-21

**Authors:** Mihai Popescu, Mihaela Roxana Olita, Mara Oana Stefan, Mariana Mihaila, Romina-Marina Sima, Dana Tomescu

**Affiliations:** 1 Department of Anaesthesia and Critical Care, Fundeni Clinical Institute, Carol Davila University of Medicine and Pharmacy, 258 Fundeni Street, 2nddistrict, 022328 Bucharest, Romania; 2 Department of Anaesthesia and Critical Care III, Fundeni Clinical Institute, Bucharest, Romania; 3 Department of Internal Medicine, Fundeni Clinical Institute, Bucharest, Romania; 4 Department of Obstetrics and Gynecology, Bucur Maternity, Carol Davila University of Medicine and Pharmacy, Bucharest, Romania

**Keywords:** Video-assisted surgery, Laparoscopy, Mechanical ventilation, Lung mechanics

## Abstract

**Background:**

Video-assisted surgery has become an increasingly used surgical technique in patients undergoing major thoracic and abdominal surgery and is associated with significant perioperative respiratory and cardiovascular changes. The aim of this study was to investigate the effect of intraoperative pneumoperitoneum during video-assisted surgery on respiratory physiology in patients undergoing robotic-assisted surgery compared to patients undergoing classic laparoscopy in Trendelenburg position.

**Methods:**

Twenty-five patients undergoing robotic-assisted surgery (RAS) were compared with twenty patients undergoing classic laparoscopy (LAS). Intraoperative ventilatory parameters (lung compliance and plateau airway pressure) were recorded at five specific timepoints: after induction of anesthesia, after carbon dioxide (CO_2_) insufflation, one-hour, and two-hours into surgery and at the end of surgery. At the same time, arterial and end-tidal CO_2_ values were noted and arterial to end-tidal CO_2_ gradient was calculated.

**Results:**

We observed a statistically significant difference in plateau pressure between RAS and LAS at one-hour (26.2 ± 4.5 cmH_2_O vs. 20.2 ± 3.5 cmH_2_O, *p* = 0.05) and two-hour intervals (25.2 ± 5.7 cmH_2_O vs. 17.9 ± 3.1 cmH_2_O, *p* = 0.01) during surgery and at the end of surgery (19.9 ± 5.0 cmH_2_O vs. 17.0 ± 2.7 cmH_2_O, *p* = 0.02). Significant changes in lung compliance were also observed between groups at one-hour (28.2 ± 8.5 mL/cmH_2_O vs. 40.5 ± 13.9 mL/cmH_2_O, *p* = 0.01) and two-hour intervals (26.2 ± 7.8 mL/cmH_2_O vs. 54.6 ± 16.9 mL/cmH_2_O, *p* = 0.01) and at the end of surgery (36.3 ± 9.9 mL/cmH_2_O vs. 58.2 ± 21.3 mL/cmH_2_O, *p* = 0.01). At the end of surgery, plateau pressures remained higher than preoperative values in both groups, but lung compliance remained significantly lower than preoperative values only in patients undergoing RAS with a mean 24% change compared to 1.7% change in the LAS group (*p* = 0.01). We also noted a more significant arterial to end-tidal CO_2_ gradient in the RAS group compared to LAS group at one-hour (12.9 ± 4.5 mmHg vs. 7.4 ± 4.4 mmHg, *p* = 0.02) and two-hours interval (15.2 ± 4.5 mmHg vs. 7.7 ± 4.9 mmHg, *p* = 0.02), as well as at the end of surgery (11.0 ± 6.6 mmHg vs. 7.0 ± 4.6 mmHg, *p* = 0.03).

**Conclusion:**

Video-assisted surgery is associated with significant changes in lung mechanics after induction of pneumoperitoneum. The observed changes are more severe and longer-lasting in patients undergoing robotic-assisted surgery compared to classic laparoscopy.

**Supplementary Information:**

The online version contains supplementary material available at 10.1186/s12871-022-01900-5.

## Introduction

Video assisted surgery (VAS) has become extensively used worldwide in cardiothoracic and major abdominal surgery, including gynecological and urological pro-cedures [1, 2]. VAS, in combination with early-recovery after surgery protocols, enhances patient recovery, lowers overall costs, and shortens hospital stay with the same oncologic outcomes as laparotomy [3–5]. As patients presenting for VAS are becoming older and with more severe co-morbidities anesthesiologists are presented with new challenges and cases are becoming more difficult to manage. Trends in anesthesia for VAS are changing and anesthesiologists must provide both proper anesthesia to facilitate the surgical technique [6] and to assure patient safety throughout the perioperative period, minimizing perioperative risks [7].

From an anesthesiologists’ point of view the main problems during VAS are related to patient positioning and pneumoperitoneum induced changes in cardiovascular and respiratory physiology. Insufflation of carbon dioxide (CO_2_) during VAS is associated with an increase in mean arterial pressure and systemic vascular resistance and a decrease cardiac output [8] and renal blood flow [9]. In the pulmonary system, pneumoperitoneum is associated with an increase in plateau pressure and a decrease in lung compliance making mechanical ventilation and effective CO2 removal a potential problem during surgery [10].

Two VAS techniques are generally used during major abdominal surgery: classic laparoscopic surgery (LAS) or robotic assisted surgery (RAS). Although most studies demonstrate similar surgical outcomes between the two techniques [11], no study to date has focused on the comparative effects of LAS and RAS on pulmonary mechanics. The primary outcome was to assess the effects of pneumoperitoneum on lung compliance and airway pressure during video-assisted surgery (VAS) in patients undergoing RAS compared to patients undergoing LAS in Trendelenburg position. The secondary objective was to assess the effect of this changes on arterial to end-tidal CO_2_ gradient and the return of both lung compliance and airway pressure to pre-pneumoperitoneum values at the end of surgery.

## Methods

The ethical approval for the present study was provided by the Ethical Committee of Fundeni Clinical Institute, Bucharest, Romania, and all patients signed the informed consent.

Twenty-five consecutive patients who underwent RAS were matched based on age, body mass index, American Society of Anesthesiologists (ASA) score, duration of surgery and of pneumoperitoneum and baseline lung mechanics parameters (Table 1) with a second group of twenty patients who underwent LAS in the Department of General Surgery and Liver Transplantation at Fundeni Clinical Institute. The decision to perform either RAS or LAS was made by the attending surgeon prior to patient inclusion. All patients underwent VAS in Trendelenburg position for endometrial cancer or rectal carcinoma. Exclusion criteria consisted of age under 18 years, a body mass index > 35 kg/m^2^, conversion of VAS to laparotomy and severe preoperative pulmonary or cardiovascular co-morbidities.

**Table 1:** Patient characteristics

Patient characteristics	RAS group (*n* = 25)	LAS group (*n* = 20)	*P* value
Age (years)	61.0 ± 11.9	59.9 ± 15.1	0.54
BMI (kg/m^2^)	27.9 ± 6.1	25.4 ± 6.4	0.82
ASA III status	100%	100%	0.99
Duration of surgery (min)	266 ± 84	205 ± 50	0.17
Duration of pneumoperitoneum (min)	207 ± 77	164 ± 35	0.22
Pplat (cm H_2_O)	15.1 ± 3.4	14.9 ± 2.7	0.11
Lc (mL/cmH_2_O)	48.1 ± 8.8	58.0 ± 9.8	0.77
PaCO_2_ (mmHg)	40.0 ± 5.4	35.1 ± 4.7	0.52
ΔCO_2_ (mmHg)	4.6 ± 2.1	5.0 ± 1.8	0.41

*BMI* Body mass index, ASA – American Society of Anesthesiologists Classification, Pplat – plateau pressure, *Lc* Lung compliance, *PaCO*_*2*_ Arterial partial pressure of CO_2_, *ΔCO*_*2*_ arterial to end-tidal CO_2_ gradient

All surgeries were performed under general anesthesia by four surgeons experienced in VAS. Induction of anesthesia was performed using propofol, fentanyl and atracurium and maintained of anesthesia was achieved with Sevoflurane and fentanyl. Subsequent doses of atracurium were administered during surgery guided by train of four monitoring. The lungs were ventilated using a Perseus A500 Anesthesia Machine (Dräger Medical®, Lübeck, Germany) in a volume-controlled mode with an oxygen/air mixture of 0.5. Ventilator settings were tidal volume of 7 mL/kg, inspiratory/expiratory ratio 1:2, an inspiratory fresh gas flow of 2.0 L/min and an end-expiratory positive pressure of 5 mmHg. Respiratory rate was adjusted to maintain an EtCO2 pressure of 36 ± 4 mmHg. An arterial catheter was inserted on the radial artery before induction of anesthesia for blood sample collection and hemodynamic monitoring. Pneumoperitoneum was obtained by CO2 insufflation after induction of anesthesia and was automatically maintained at 12–14 mmHg in both groups.

Patient age, sex, height, and weight were collected by the attending anesthesiologist before surgery. Arterial blood samples and ventilatory parameters were obtained at five specific time points: after induction of anesthesia (T0), after induction of pneumoperitoneum (T1), one-hour into surgery (T2), two-hours into surgery (T3) and at the end of surgery (T4). Arterial blood gases analysis was performed on ABL 800 Radiometer (Medical APS®, Brǿnshǿj, Denmark). Lung compliance (Lc) was defined as pulmonary compliance during periods without gas flow, such as during an inspiratory pause. The following ventilatory parameters were recorded at the five timepoints: plateau airway pressure (Pplat), Lc after performing an inspiratory hold maneuver and end-tidal CO_2_ (EtCO_2_). The arterial to EtCO_2_ gradient (ΔCO_2_) was calculated as the arithmetic difference between the measured arterial oxygen pressure (PaO_2_) and the mean EtCO_2_ during the minute before obtaining the arterial blood sample. Percentual change in either Lc or Pplat was calculated by the following formula: [(parameter at a time “x” – parameter at time “x + 1”)/parameter at time “x”] *100 and results were recorded as absolute values. Haemodynamic variables, mean arterial blood pressure measured invasively – MAP and heart rate – HR, were recorded at the same time points.

In order to detect clinically significant 15% change in lung compliance and airway pressure, based on mean variables cited in the literature, 24 patients were included in the RAS group in order to obtain a 75% statical power. The 15% change was based on previously published data from our study group, as well as that demonstrated by other studies [10, 12] These patients were matched on a 0.8 ratio to 20 patients undergoing LAS. Statistical analyses were performed using SPSS 19.0 (SPSS Inc®, Chicago, IL, USA). Data are presented as mean ± standard deviation of the mean. Data distribution was examined for normality using Kolmogorov Smirnov test to insure the proper statistical examination. Categorical variables were analyzed by utilizing the Chi-square test and quantitative data were analyzed with independent samples t-test. Mann–Whitney test was used when the analyzed data did not follow a normal distribution. All P values are two-tailed and a *P* value ≤ 0.05 was considered statistically signdicant.

## Results

Twenty-five patients were included in the RAS group and twenty patients in the LAS group. No statistically significant differences regarding preoperative variables were identified between the two groups (Table 1). Pplat increased and Lc decreased after the induction of pneumoperitoneum, but no significant difference was observed between the two groups. We observed a statistically significant difference in Pplat and Lc between RAS and LAS at one-hour and two-hour intervals during surgery and at the end of surgery. Data are presented in Table 2.

**Table 2:** Comparison of ventilatory parameters and arterial to end-tidal CO2 gradient between the two groups

Time of measurement	Patient parameters	RAS group (*n* = 25)	LAS group (*n* = 20)	*P* value
Postinduction of Pneumoperitoneum	Pplat (cmH_2_O)	25.0 ± 5.3	22.2 ± 4.6	0.75
	Lc (mL/cmH_2_O)	26.4 ± 6.4	33.0 ± 7.2	0.33
	ΔCO_2_ (mmHg)	7.9 ± 3.9	6.1 ± 4.5	0.36
One-hour into surgery	Pplat (cmH_2_O)	26.2 ± 4.5	20.2 ± 3.5	0.05*
	Lc (mL/cmH_2_O)	28.2 ± 8.5	40.5 ± 13.9	0.01*
	ΔCO_2_ (mmHg)	12.9 ± 4.5	7.4 ± 4.4	0.02*
Two-hour into surgery	Pplat at T3 (cmH_2_O)	25.2 ± 5.7	17.9 ± 3.1	0.01*
	Lc at T3 (mL/cmH_2_O)	26.2 ± 7.8	54.6 ± 16.9	0.01*
	ΔCO_2_ (mmHg)	15.2 ± 4.5	7.7 ± 4.9	0.02*
End of surgery	Pplat at T4 (cmH_2_O)	19.9 ± 5.0	17.0 ± 2.7	0.02*
	Lc at T4 (mL/cmH_2_O)	36.3 ± 9.9	58.2 ± 21.3	0.01*
	ΔCO_2_ (mmHg)	11.0 ± 6.6	7.0 ± 4.6	0.03*

*Pplat* Plateau pressure, *Lc* Lung compliance, *ΔCO2* Arterial to end-tidal CO2 gradient

**Fig. 1:**
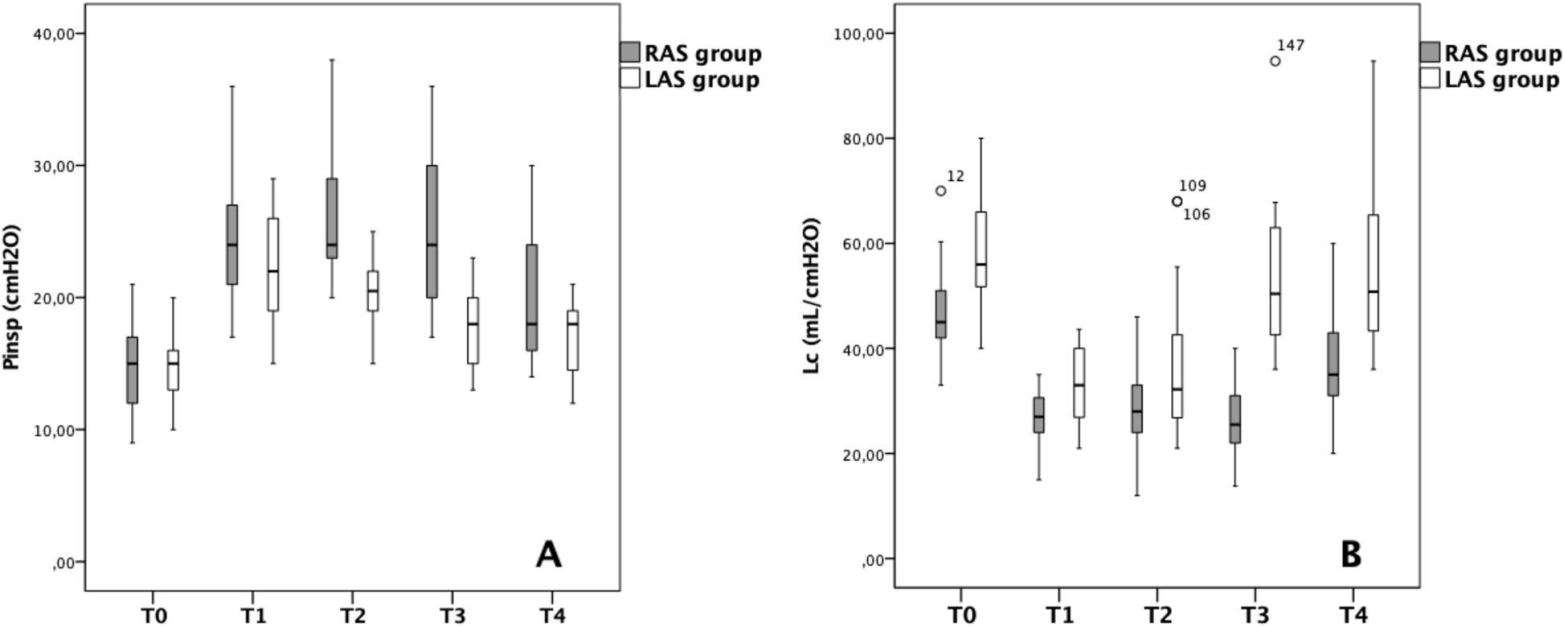
Comparison of plateau pressure (Pplat) – **A** and lung compliance (Lc) – **B** between patients undergoing robotic-assisted surgery (RAS) and classic laparoscopy (LAS)

Pplat significantly increased to 25.0 ± 5.3 cmH_2_O in the RAS group after induction of pneumoperitoneum compared to 15.1 ± 3.4 cmH_2_O postinduction of anesthesia (*p* = 0.05) but did not change compared to this level at both one-hour (26.2 ± 4.5 cmH_2_O, *p* = 0.48) and two-hours into surgery (25.2 ± 5.7 cmH_2_O, *p* = 0.11) or at the end of surgery (19.9 ± 5.0 cmH_2_O, *p* = 0.36). Pplat remained significantly higher at the end of surgery compared to postinduction of anesthesia (*p* = 0.05). In the LAS group, Pplat increased to 22.2 ± 4.6 cmH_2_O after induction of pneumoperitoneum compared to 14.9 ± 2.7 cmH_2_O postinduction of anesthesia (*p* = 0.01) but did not change intraoperatively at one-hour (20.2 ± 3.5 cmH_2_O, *p* = 0.28) and two-hours into surgery (17.9 ± 3.1 cmH_2_O, *p* = 0.59) or at the end of surgery (17.0 ± 2.7 cmH_2_O, *p* = 0.69). Pplat remained significantly higher at the end of surgery compared to postinduction of anesthesia (*p* = 0.02)—Fig. 1A.

In the RAS group, Lc decreased significantly to 26.4 ± 6.4 mL/cmH_2_O after insufflation of pneumoperitoneum compared to 48.1 ± 8.8 mL/cmH_2_O postinduction of anesthesia (*p* = 0.03) but did not significantly change com-pared to this level at one-hour (28.2 ± 8.5 mL/cmH_2_O, *p* = 0.104) or two-hours into surgery (26.2 ± 7.8 mL/cmH_2_O, *p* = 0.71) or between the second hour and the end of surgery (36.3 ± 9.9 mL/cmH_2_O, *p* = 0.43). Lc remained statistically significantly lower at end of anesthesia compared postinduction of anesthesia (*p* = 0.04). In the LAS group, Lc decreased significantly to 33.0 ± 7.2 mL/cmH_2_O after induction of pneumoperitoneum compared to 58.0 ± 9.8 mL/cmH_2_O postinduction of anesthesia (*p* = 0.02), then significantly increased at one-hour into surgery compared to postinduction of pneumoperitoneum (40.5 ± 13.9 mL/cmH_2_O, *p* = 0.02) and remained constant at two-hours into surgery (54.6 ± 16.9 cL/cmH_2_O, *p* = 0.90) and at the end of surgery (58.2 ± 21.3 mL/cmH_2_O,,*p* = 0.91). There was no statistical difference in Lc at the end of surgery compared to postinduction of anesthesia (*p* = 0.23) – Fig. 1B. The difference in percentual change in Lc and Pplat between the two groups at the five specific timepoints are presented in Table 3.

**Table 3:** Dynamic changes in lung compliance and plateau pressure

Time of measurement	Patient parameters	RAS group (*n* = 25)	LAS group (*n* = 20)	*P* value
T0 to T1	ΔPplat (%)	69.3 ± 36.5	51.6 ± 34.5	0.78
	ΔLc (%)	45.2 ± 10.4	41.8 ± 15.8	0.08
T1 to T2	ΔPplat (%)	5.9 ± 11.3	6.4 ± 15.9	0.02*
	ΔLc (%)	6.6 ± 16.1	19.0 ± 22.1	0.01*
T2 to T3	ΔPplat (%)	9.1 ± 11.1	14.0 ± 14.3	0.48
	ΔLc (%)	2.2 ± 13.2	63.2 ± 47.5	0.01*
T3 to T4	ΔPplat (%)	16.3 ± 15.8	2.6 ± 4.3	0.01*
	ΔLc (%)	38.2 ± 26.1	12.3 ± 10.1	0.01*
T4 to T0	ΔPplat (%)	34.1 ± 36.9	15.9 ± 21.9	0.02*
	ΔLc (%)	24.0 ± 15.4	1.7 ± 41.3	0.01*

*Pplat* Plateau pressure, *Lc* Lung compliance

We observed a non-significant difference between the RAS and LAS groups in ΔCO_2_ after induction of anesthesia (4.6 ± 2.1 mmHg vs. 5.0 ± 1.8 mmHg, *p* = 0.41) and after induction of pneumoperitoneum (7.9 ± 3.9 mmHg vs. 6.1 ± 4.5 mmHg, *p* = 0.36) and a statistically significant difference one-hour (12.9 ± 4.5 mmHg vs. 7.4 ± 4.4 mmHg, *p* = 0.02) and two-hours into the surgery (15.2 ± 4.5 mmHg vs. 7.7 ± 4.9 mmHg, *p* = 0.02) and at the end of surgery (11.0 ± 6.6 mmHg vs. 7.0 ± 4.6 mmHg, *p* = 0.03). Data are presented in Table 2. No significant differences were observed between the two groups in terms of measured hemodynamic parameters (MAP and HR) and PaO_2_ during the same time points. Data are presented in supplementary table 1.

## Discussion

Our results show that induction of pneumoperitoneum is associated with an increase in plateau pressure and decrease in lung compliance in patients undergoing VAS in Trendelenburg position independent of surgical technique. Our results are in accordance with previously published data in patients undergoing pelviscopic surgery [13].

The increase in Pplat during surgery observed in both groups may be explained by the increase in intra-abdominal pressure due to induction of pneumoperitoneum and Trendelenburg position that cause an upward shift of the diaphragm. This increases intrathoracic pressure and is responsible for observed changes in the distribution of ventilation and subsequent increase in ventilation-perfusion mismatch [14, 15]. However, in our study group, the Pplat failed to return to preoperative levels at the end of surgery after pneumoperitoneum was released. This is in accordance with the study published by Lian et al. [16] who showed that peak pressures remain higher than perioperative values in patients undergoing laparoscopic hysterectomy regardless of the ventilation mode applied and this may be attributed to retention of secretions and basal atelectasis that persist after pneumoperitoneum is released.

In a study by Choi et al. [17], patients who had a peak airway pressure ≥ 30 cm H_2_O had a fivefold greater incidence of postoperative respiratory complications, longer postanesthesia care unit stays, greater alveolar dead space-to-tidal volume ratios and a lower arterial partial pressure of oxygen. We consider that anesthetic strategies aimed at lowering airway pressure below this threshold are important to improve both intraoperative respiratory function and to decrease the incidence of postoperative complications. In another study, Sroussi et al. [18] showed that the use of a new insufflation system that uses a lower intra-abdominal pressure is associated with improved hemodynamics and lower peak lower pressures. The development of such surgical techniques may be useful in lowering the effects of pneumoperitoneum on lung mechanics while maintaining adequate surgical access.

The changes observed in lung compliance were more long-lasting during RAS. In this group we observed that Lc decreased after induction of pneumoperitoneum, remained low throughout surgery, and did not return to preoperative values at the end of surgery. By comparison, in the LAS group Lc decreased after induction of pneumoperitoneum, gradually increased during surgery and there was no statistically significant difference between preoperative and end-of surgery values. The decrease in Lc is mostly due to basal atelectasis, decrease in functional residual capacity and a decrease in diaphragmatic excursion during pneumoperitoneum [19]. Application of positive end-expiratory pressure may slightly recover the ventilation-perfusion mismatch in the Trendelenburg position and improve both oxygenation and lung mechanics [20]. However, studies did not find an appropriate level of positive end-expiratory pressure to improve intraoperative ventilation and lower the incidence of postoperative hypoxia [21] and further research is still needed.

Although in our study there was no significant difference in terms of length of surgery, this may represent a reason for the persistence of decreased Lc and increased Pplat at the end of surgery.

The observed differences in both Lc and Pplat between RAS and LAS were statistically significant with better lung mechanics in patients undergoing classic laparoscopy. Two main reasons can be responsible for the observed changes. The first would be a much higher insufflation pressure to maintain pneumoperitoneum during surgery. However, no difference between intraabdominal pressure was observed between the two groups. (12–14 mmHg). The second reason would be a steeper Trendelenburg position applied during RAS to improve surgical access [22]. In a study published by Mitsuhashi et al. [23] even a slightly higher increase of 5°, from 20° to 25°, in head-down position was associated with a significant increase in airway pressures in obese patients undergoing robotic-assisted hysterectomy. Interesting, in a recent survey on randomly selected active members of the American Society of Anesthesiology, more than two-thirds did not limit the duration or inclination angle during VAS [24]. This crucial of patient positioning is mostly decided by surgeons in order to improve surgical access, especially in RAS where the robustness of the device may impose a steeper position up to 45° in order to facilitate arm movement [25, 26]. From an anesthesiologic point of view, pulmonary changes associated with induction of pneumoperitoneum may be different between laparoscopic and robotic-assisted surgery and a more personalized, patient-based approach should be applied to improve lung mechanics.

One of the most important aspects of any observed physiological changes during anesthesia is the impact on patient outcome. In a recently published systematic review, Katayama et al. [27] found no correlation between steep Trendelenburg position and incidence of cardiac, cerebrovascular complications, as well as an increased risk of venous thromboembolism. However, a third of patients may experience postoperative pulmonary complications that require admission to an intensive care unit [28] and, hence, appropriate intraoperative management and correction of ventilatory alterations becomes a crucial issue. Intraoperative recruitment maneuvers and the addition of positive end-expiratory pressure (PEEP) are well documented in the literature. Kudoh et al. [29], demonstrated a significant increase in lung compliance after performing a 30 s recruitment maneuver of sustained inflation to 30 cmH_2_O alongside applying a PEEP of 5 cmH_2_O. However, due to the low number of patients we cannot assess if this is sufficient to decrease the incidence of postoperative pulmonary complications. The use of recruitment maneuvers has also been investigated in a meta-analysis published by Pei et al. [30]. Their results show a significant impact in improving intraoperative lung mechanics and reducing postoperative pulmonary complications but the exact effect of such a technique on cardiocirculatory physiology, and especially venous return and cardiac output needs further research. Using higher PEEP values was assessed by Shono et al. [31] in a randomized control trial and have demonstrated that the application of 15 cm H_2_O of PEEP resulted in a better ventilation profile and favorable physiologic effects during RAS prostatectomy_,_ however this did not improve postoperative lung function.

The mode of mechanical ventilation may also represent a key factor in lung mechanics during VAS. When comparing pressure-controlled ventilation to volume controlled-ventilation, pressure-control was associated with higher Lc and lower peak airway pressure but did not have any overall advance in terms of respiratory mechanics and hemodynamics [32]. Dual-controlled ventilation may offer the combined benefits of both volume- and pressure-controlled ventilation. In their study, Park et al. [33], demonstrated that using Autoflow they were able to also decrease the peak inspiratory airway pressure. However, due to the low number of patients no estimate on the potential impact on postoperative pulmonary could be made. Based on these studies, it seems that the best strategy should combine recruitment maneuvers, optimal PEEP, and pressure-controlled ventilation. However, to date, there are no conclusive evidence to support the best anesthetic management during VAS to improve ventilatory parameters and future research is urgently needed in order to decrease the high incidence of postoperative pulmonary strategy.

The induction of pneumoperitoneum was associated with an increase in arterial to end-tidal CO_2_ difference. Absorption of CO_2_ during surgery and increased ventilation-perfusion mismatch is responsible for the higher CO_2_ gradient [34]. Kamine et al. [35] showed that higher abdominal pressure values were associated with decreased end-tidal CO_2_ values. Although abdominal pressure was identical between the two groups, we observed that patients in the RAS group had both a higher CO_2_ gradient and a decreased lung compliance. This may be related with increased atelectasis and a higher shunt fraction that is responsible for the difference in arterial to end-tidal CO_2_, and thus making ΔCO_2_ a useful marker in the assessment of ventilation-perfusion mismatch.

The present study has some limitations. First, this was an observational, retrospective, single-center study and all patients received the same ventilatory strategy independent of the video-assisted technique use and so, the observed difference in ventilatory mechanics, may be minimized by a more personalized approach on ventilation and positive end-expiratory pressure titration. The authors are aware of the fact that these strategies can very between centers and our remarks may apply only in patients who undergo VAS under similar conditions of mechanical ventilation. Secondary, some parameters, like the steepness of Trendelenburg position and shunt fraction, that may have an important effect on lung physiology, could not be assessed. Thirdly, the low number of patients was insufficient to assess the effects of intraoperative lung mechanics on postoperative outcome. Future studies are needed to investigate the composite effect of surgical position, type of VAS used and intraoperative recruitment maneuvers on perioperative lung mechanics.

## Conclusion

In conclusion VAS, regardless of whether RAS or LAS was used, or is associated with increased airway pressure and decreased lung compliance. The effects of pneumoperitoneum on lung mechanics are more pronounced in patients undergoing robotic-assisted surgery compared to classic laparoscopy. Although airway pressures failed to return to preoperative values in both groups at the end of surgery, changes in lung compliance were minimal compared to preoperative values in patients undergoing classic laparoscopy compared to RAS after the pneumoperitoneum was released. The decrease in lung compliance and increase in plateau pressure was associated with a greater arterial to end-tidal CO_2_ gradient.

## Supplementary Information

**Additional file 1** **Supplementary Table 1. **Comparison of hemodynamics parameters and PaO_2_ between the two groups.

## Data Availability

The datasets used and/or analysed during the current study are available from the corresponding author on reasonable request.

## Abbreviations

VAS: Video-assisted surgery
RAS: Robotic-assisted surgery
LAS: Classic laparoscopic surgery
ASA score: American Society of Anesthesiologists score
CO_2_: Carbon dioxide
EtCO_2_: End-tidal carbon dioxide
PaO_2_: Arterial partial pressure of oxygen
Lc: Lung compliance
Pplat: Plateau pressure
