# Supplementary material for: Lung mechanics during video-assisted abdominal surgery in Trendelenburg position: a cross-sectional propensity-matched comparison between classic laparoscopy and robotic-assisted surgery
Source: BMC Anesthesiol. 2022 Nov 21;22:356. doi: 10.1186/s12871-022-01900-5 (PMC9677621; doi:10.1186/s12871-022-01900-5)
Supplement: Supplementary file 1 — Additional file 1 Supplementary Table 1. Comparison of hemodynamics parameters and PaO2 between the two groups. [file 12871_2022_1900_MOESM1_ESM.docx]

| **Time of measurement** | **Patient parameters** | **RAS group**  **(n=25)** | **LAS group**  **(n=20)** | **P value** |
| --- | --- | --- | --- | --- |
| Postinduction of Pneumoperitoneum | MAP (mmHg) | 83±9 | 83±11 | 0.78 |
|  | HR (beats/minute) | 78±8 | 74±8 | 0.80 |
|  | PaO_2_ / FiO_2_ | 419±43 | 428±41 | 0.81 |
| One-hour into surgery | MAP (mmHg) | 72±4 | 74±6 | 0.17 |
|  | HR (beats/minute) | 76±10 | 81±10 | 0.66 |
|  | PaO_2_ / FiO_2_ | 399±53 | 404±49 | 0.81 |
| Two-hour into surgery | MAP (mmHg) | 73±6 | 72±4 | 0.0.38 |
|  | HR (beats/minute) | 75±6 | 74±7 | 0.38 |
|  | PaO_2_ / FiO_2_ | 360±39 | 370±35 | 0.82 |
| End of surgery | MAP (mmHg) | 73±5 | 73±4 | 0.63 |
|  | HR (beats/minute) | 74±7 | 76±7 | 0.63 |
|  | PaO_2_ / FiO_2_ | 340±30 | 345±24 | 0.28 |

**Supplementary Table 1.** Comparison of hemodynamics parameters and PaO_2_ between the two groups.

Legend. MAP – mean arterial pressure, HR – heart rate, PaO_2_ – arterial partial pressure of oxygen, FiO_2_ – inspiratory fraction of oxygen
